# 3′3-Diindolylmethane inhibits migration, invasion and metastasis of hepatocellular carcinoma by suppressing FAK signaling

**DOI:** 10.18632/oncotarget.4196

**Published:** 2015-06-03

**Authors:** Wen-Xue Li, Li-Ping Chen, Min-Ying Sun, Jun-Tao Li, Hua-Zhang Liu, Wei Zhu

**Affiliations:** ^1^ Dearpartmant of Toxicology, Guangzhou Center for Disease Control and Prevention, Guangzhou, China; ^2^ Faculty of Toxicology, School of Public Health, Sun Yet-sen University, Guangzhou, China

**Keywords:** hepatocellular carcinoma, focal adhesion kinase (FAK), diindolylmethane (DIM), MMP2/9, pTEN

## Abstract

Late stage hepatocellular carcinoma (HCC) usually has a low survival rate because it has high potential of metastases and there is no effective cure. 3′3-Diindolylmethane (DIM) is the major product of the acid-catalyzed oligomerization of indole-3-carbinol present in cruciferous vegetables. DIM has been proved to exhibit anticancer properties. In this study, we explored the effects and molecular mechanisms of anti-metastasis of DIM on HCC cells both *in vitro* and *in vivo*. We chose two HCC cell lines SMMC-7721 and MHCC-97H that have high potential of invasion. The results showed that DIM inhibited the proliferation, migration and invasion of these two cell lines *in vitro*. In addition, *in vivo* study demonstrated that DIM significantly decreased the volumes of SMMC-7721 orthotopic liver tumor and suppressed lung metastasis in nude mice. Focal Adhesion Kinase (FAK) is found over activated in HCC cells. We found that DIM decreased the level of phospho-FAK (Tyr397) both *in vitro* and *in vivo*. DIM inhibition of phospho-FAK (Tyr397) led to down-regulation of MMP2/9 and decreased potential of metastasis. DIM also repressed the migration and invasion induced by vitronectin through inactivation of FAK pathway and down-regulation of MMP2/9 *in vitro*. We also found that pTEN plays a role in down-regulation of FAK by DIM. These results demonstrated that DIM blocks HCC cell metastasis by suppressing tumor cell migration and invasion. The anti-metastasis effect of DIM could be explained to be its down-regulated expression and activation of MMP2/9 partly induced by up-regulation of pTEN and inhibition of phospho-FAK (Tyr397).

## INTRODUCTION

Hepatocellular carcinoma(HCC) is one of the most common and aggressive human malignancies around the world, especially in developing countries [1]. Although great progress was attained in cancer treatment, most HCC patients die from cancer invasion or distant metastasis to other organs. Therefore, preventing metastasis of cancer cells is one of the effective strategies for the successful management of HCC.

The mechanism of cancer invasion and metastasis is a complicated multistep process involving multiple genetic alterations. Focal Adhesion Kinase (FAK), a 125 kD non-receptor protein tyrosine kinase, is the key signaling nexus connecting integrins and the dynamic actin cytoskeleton to coordinate cell motility and cell invasion [2]. Interaction between integrins and extracellular matrix(ECM) lead to the activation of FAK through phosphorylation and autophosphorylation at the tyrosine 397(Tyr397). The activation of FAK is the key regulatory point for several signaling pathways. Activated FAK interacts with PI3 kinase (PI3K)-dependent Akt (PI3K/AKT) axis to interrupt apoptotic pathways and increase tumor cell migration [3–5]. Activated FAK regulates survival and metastasis signaling pathways through PI3K/AKT pathway and downstream signaling including MMPs and IAPs activation in HCC [6, 7]. Both FAK and phosphorylated FAK Tyr397 have been shown to be overexpressed in HCC samples and HCC cell lines. In addition, increased FAK and phospho-FAK (Tyr397) expression were correlated with tumor stage, vascular invasion and intrahepatic metastasis in HCC [2]. These data suggest that deregulation of FAK plays an important role in HCC malignant progression. Many ways for inhibition of FAK including siRNA and small molecule inhibitors has been shown to decrease cellular migration and invasion in multiple tumor types, including HCC [8–10]. Therefore, inactivation of FAK pathway could be a good way to inhibit metastasis of human HCC.

A number of studies demonstrate that diets rich in fruits and vegetables could reduce the risk of cancers through some kinds of promising anti-cancer phytochemicals in them [11]. One of the potential phytochemicals is 3′3-Diindolylmethane (DIM). DIM is the predominant active product of the acid-catalyzed oligomerization of indole-3-carbinol (I3C), a phytochemical from vegetables of the family cruciferous [12]. DIM has been reported to reduce carcinogen-induced breast and lung tumor formation in rodent models [13, 14]. The growth of bladder and breast cancer xenografts were also shown to be inhibited by DIM administration [15]. It has also been showed that DIM blocks the metastasis of many types of cancer cells both *in vitro* and *in vivo*, including breast cancer [16], prostate cancer [17, 18], lung cancer [16] and bladder cancer [19]. However, the mechanism underlying the role of DIM on HCC treatment still remains elusive. We initiated this study to investigate the anti-cancer properties of DIM and elucidate the underlying mechanisms.

In this study, we investigated the role of DIM in the anti-metastasis effect on HCC cell lines SMMC-7721 and MHCC-97H. These cells have high potential of invasion. We found that DIM dose-dependently inhibited proliferation, migration, adhesion and invasion of SMMC-7721 and MHCC-97H *in vitro*. Oral administration of DIM inhibited lung metastasis of SMMC-7721 xenograft in BALB/c nude mice. Furthermore, we demonstrate that DIM increased the expression of pTEN and inhibited the phosphorylation of FAK Tyr397, in succession led to the down-regulation of MMP2/9.

## RESULTS

### DIM significantly inhibited the proliferation of HCC cells

**Figure 1:**
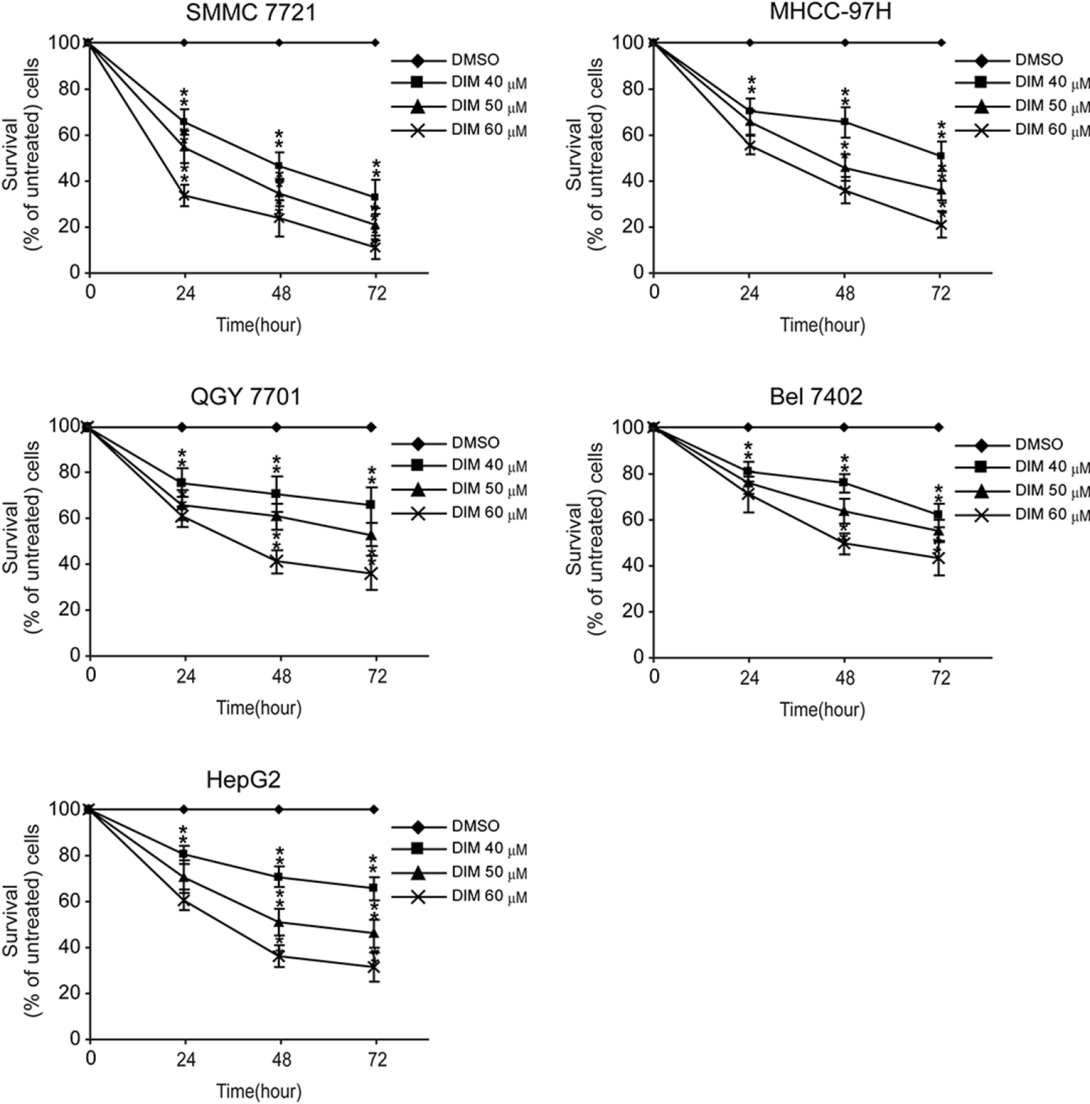
Effects of DIM on the proliferation of HCC cells SMMC-7721, MHCC-97H, QGY-7701, Bel-7402 and HepG2 cells were treated without or with increasing concentration of DIM (30, 40 and 60 μM for 24, 48 and 72 h). After treatment, WST-1 was added and incubated for 2 h at 37°C. Light absorbance was recorded at 450nM. Inhibition of DIM on cell proliferation was calculated based on the absorbance ration between treatment and control. Values represent mean ± SD of three independent experiments. *P < 0.05, **P < 0.01, ***P < 0.001 compared with the untreated control (dose 0).

The inhibitory effect of DIM on HCC cells was tested on HCC cell lines including SMMC-7721, MHCC-97H, QGY-7701, Bel-7402 and HepG2 cells. As shown in Fig 1, treatment with increasing concentration of DIM at 40, 50 and 60 μM for 24 h, 48 h and 72 h obviously inhibited the proliferation of these cells in a time- and dose-dependent manner. Specifically, after treatment of DIM at 40 μM for 24 h the survival rate of SMMC-7721 cells was 71%, MHCC-97H cells 75%, QGY-7701 cells 78%, Bel-7402 cells 82%, and HepG2 83% of control un-treated cells. The inhibition has statistical significance (all *P* < 0.01). With increasing the dose and time of treatment, the effect of inhibition increased accordingly. These results suggested that DIM could efficiently inhibit the proliferation of hepatocellular carcinoma cells. This result was consistent with previous works of us and others.

### FAK and MMP2/9 up-regulated in SMMC-7721 and MHCC-97H cells

**Figure 2:**
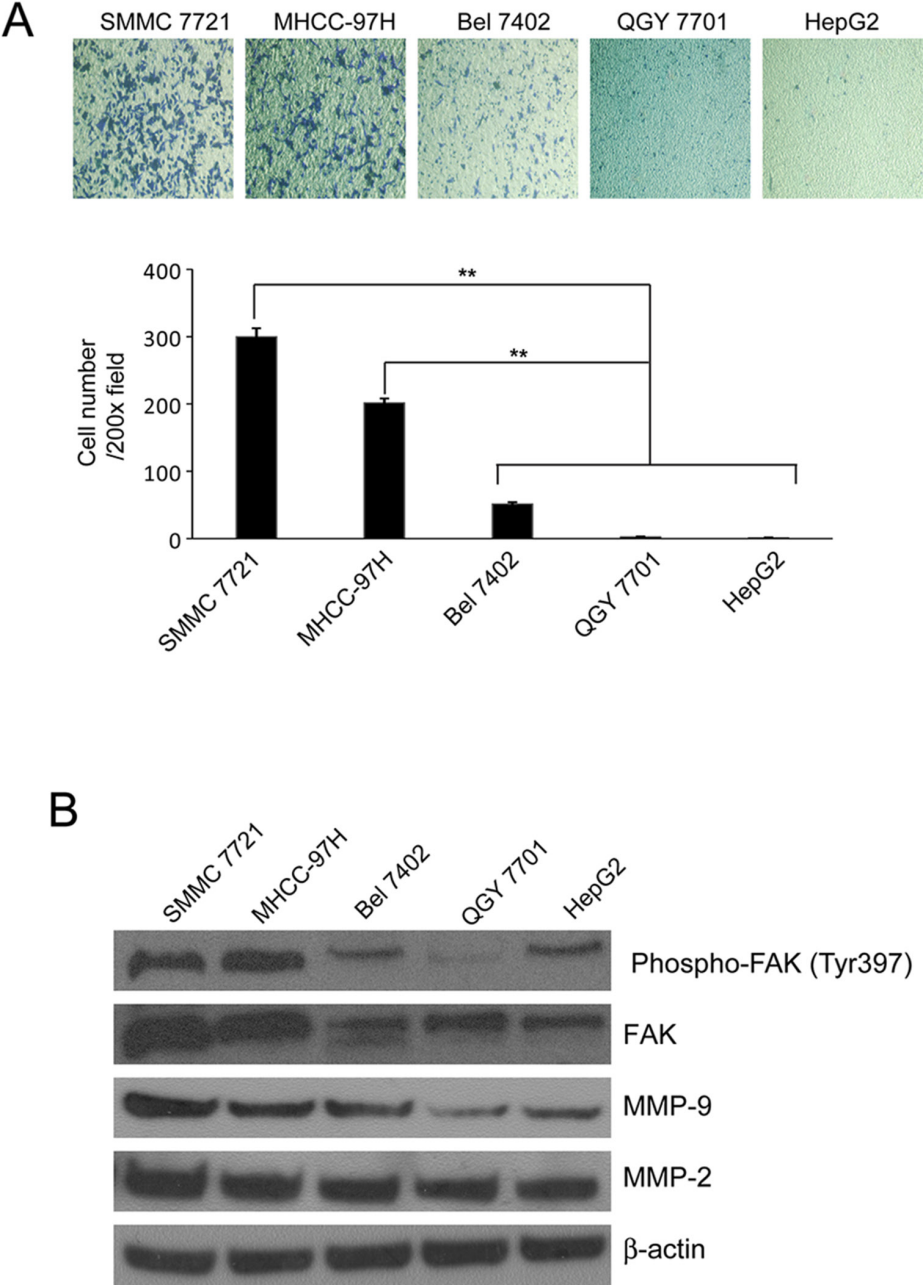
The invasiveness and the expression of FAK, phosphorylated FAK Tyr397 and MMP2/9 in HCC cells **A.** Transwell inserts were used. SMMC-7721, MHCC-97H, QGY-7701, Bel-7402 and HepG2 cells were seeded in inserts with 200 μl no-serum medium containing 1 × 10^5^ cells, 800 μl medium containing 5% FBS was added in bottom wells and cells were incubated for 24 hours and stained with Giemsa. **B.** Cells were cultured in medium containing 5% FBS and collected. The cell lysates were subjected to Western blotting analysis using antibodies against FAK, phosphorylated FAK Tyr397 and MMP2/9. β-Actin was used as loading control. Mean ± SD of three independent experiments were represented. *P < 0.05, **P < 0.01, ***P < 0.001 compared with the untreated control (dose 0).

To investigate the ability of migration and invasion of HCC cells, we used transwell assay and found that SMMC-7721 and MNCC-97H cells invaded through the transwell membrane coated with Matrigel more efficient than other cell lines as shown in Fig 2A. Previous studies show that FAK is overexpressed in HCC cell lines, and the level of FAK expression correlated with cell migration and invasion [2]. We explored the expression of FAK and phosphorylated FAK (Tyr397) in these cell lines and found that SMMC-7721 and MNCC-97H cells have higher levels of FAK and phosphorylated FAK (Tyr397) compared with other cell lines with lower potential of invasion (Fig 2B). This result was consistent with previous report [2]. Because MMP2/9 play important roles in tumor invasion and metastasis [20, 21], FAK contributes to the invasion and metastasis of HCC partly through regulating expression and activation of both MMP-2 and MMP-9 [2]. We tested the expression of MMP2/9 in these cell lines and found that there were higher expressions of MMP2/9 in SMMC-7721 and MNCC-97H cells compared with that in other cell lines (Fig 2B). Therefore, SMMC-7721 and MHCC-97H cells were chosen to be our target cells in the following steps to study the inhibitory effects of DIM on the metastasis of HCC cells.

### DIM inhibited the adhesion, migration and invasion of SMMC-7721 and MHCC-97H cells

**Figure 3:**
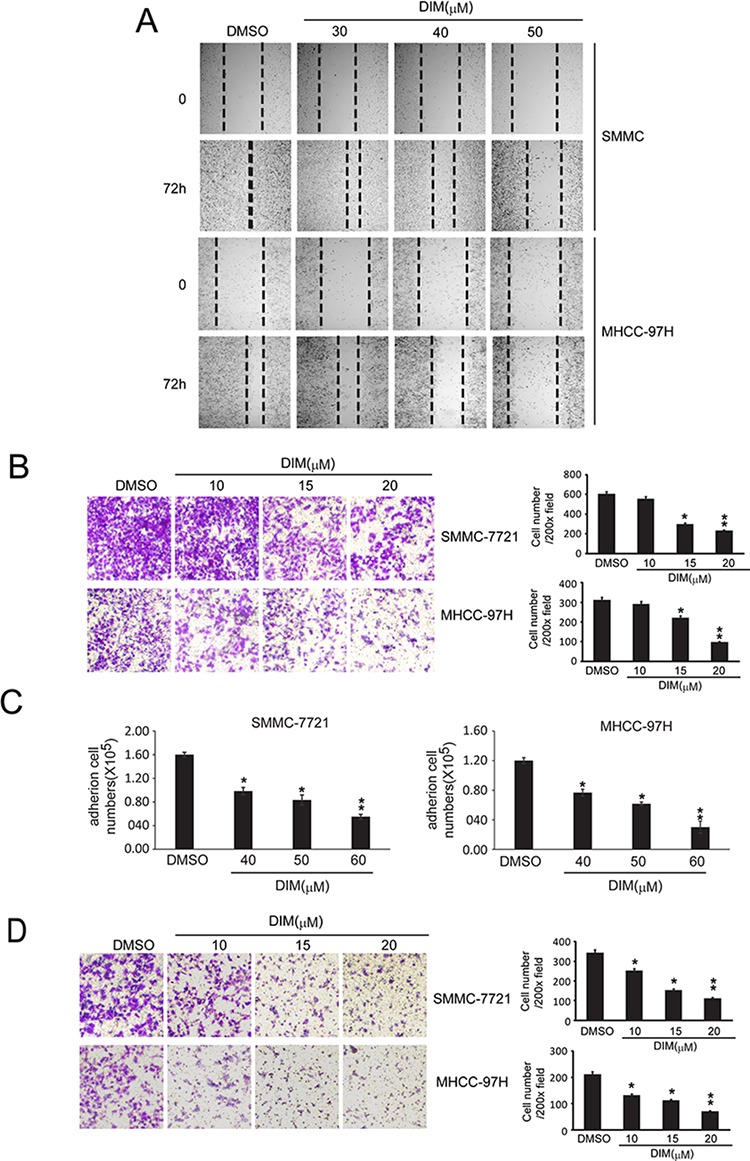
DIM inhibited the adhesion, migration and invasion of SMMC-7721 and MHCC-97H **A.** Wound healing assay were conducted. SMMC-7721 and MHCC-97H cells were seeded into 6-well plates and treated with DMSO or DIM (30 h, 40 h and 50 μM for 72 hs). Photographs were taken after treatment. **B.** Migration tests were performed by transwells inserts without basement membrane extract. SMMC-7721 and MHCC-97H (1 × 10^5^ cells per well) were seeded in insert with 200 μl no-serum medium containing different concentration of DIM (5, 10 and 15 μM), 800 μl medium containing 5% FBS was added in bottom wells and cells were incubated for 16 hours. Cells were stained with Giemsa. **C.** Cell adhesion assay. SMMC-7721 and MHCC-97H were treated in 6-well culture dishes with DIM at 30, 40 and 50 μM for 48 hours in medium with 10% FBS. After that, Cells (5 × 10^5^ cells/well) were plated in 6-well culture dishes and allowed to adhere for 2.5 h. After that, adhered cells were counted after staining with 0.4% trypan blue solution. **D.** Cells invasion assay was performed by transwell inserts with basement membrane extract. SMMC-7721 and MHCC-97H (1 × 10^5^ cells per well) were seeded in inserts with 200 μl no-serum medium containing different concentration of DIM (10, 15 and 20 μM), 800 μl medium containing 5% FBS was added in bottom wells and cells were incubated for 24 h. Cells were stained with Giemsa. Values represent mean ± SD of three independent experiments. *P < 0.05, **P < 0.01, ***P < 0.001 compared with the untreated control (dose 0).

Tumor metastasis is a dynamic hallmark of cancer which consists of three essential events; migration of cancer cells from a primary foci to secondary organs, adhesion of cancer cells at the secondary site and invasion of extracellular matrix (ECM) of secondary organ [22, 23]. We used wound healing assay to investigate the migration ability of SMMC-7721 and MHCC-97H cells. As shown in Fig 3A, we found that treatment of 30, 40 and 50 μM DIM for 72 h reduced the ability of SMMC-7721 cells to migrate from one end of wound to the other. This result was further confirmed by transwell assay. We found that DIM significantly reduced the number of SMMC-7721 and MHCC-97H cells migrating through the transwell membrane to the lower chamber in a concentration-dependent manner, in which DIM at the concentration of 15 μM decreased migrating SMMC-7721 cell number to 52% and MHCC-97H cell numbers to 67% of the control (Fig 3B). The effect of decreased migration was of statistical significance and could also be seen at the concentration of 10 μM in both cell lines. These results demonstrated that DIM could inhibit the migration of HCC cells.

The inhibitory effect of DIM on cell adhesion was also investigated on these two cell lines. The results were shown as Fig 3C. We found that DIM significantly decreased the number of cells adhered to cell culture dishes in a concentration-dependent manner. At the concentration of 50 μM, DIM decreased the adhered cell number of SMMC-7721 and MHCC-97H to 35% and 22% of control respectively (*P* < 0.01).

The inhibitory effect of DIM on invasion was investigated by examining the ability of SMMC-7721 and MHCC-97H cells cross through a simulated extracellular matrix. As shown in Fig 3D, treatment of DIM at concentrations from 10 μM to 20 μM inhibited the invasion ability of SMMC-7721 and MHCC-97H at a concentration-dependent manner. When cells were treated with DIM at 10 μM, only 77% and 50% of SMMC-7721 and MHCC-97H could invade through the simulated extracellular matrix and migrate to the insert part. In the presence of 20 μM DIM, SMMC-7721 and MHCC-97H could hardly transverse and migrate into the lower part of insert. Taken together, these results indicated that DIM acted directly on SMMC-7721 and MHCC-97H to inhibit the processes of proliferation, migration and invasion.

### DIM inhibited FAK phosphorylation and decreased the expressions and activities of MMP-2 and MMP-9

**Figure 4:**
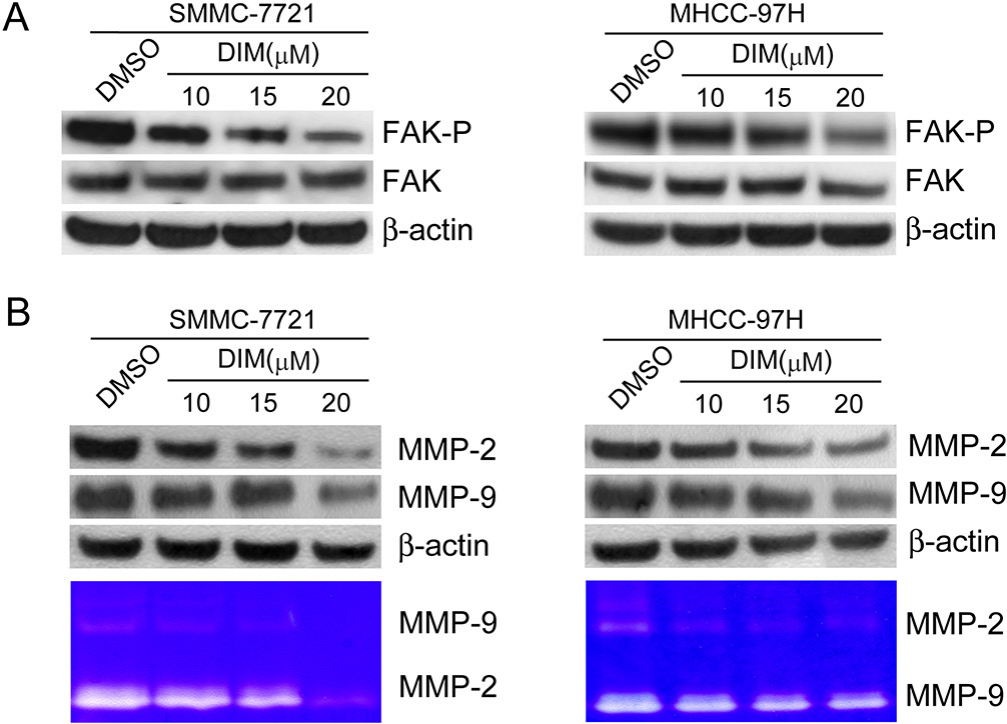
DIM inhibited FAK phosphorylation and decreased the expressions and activities of MMP-2 and MMP-9 in SMMC-7721 and MHCC-97H cells SMMC-7721 and MHCC-97H cells were treated with DIM at concentration of 10, 15 and 20 μM in serum-free medium for 48 hours. **A.** FAK and the phosphorylated FAK (Y397) was analyzed by Western blotting. β-Actin was used as loading control. **B.** Western blot and zymography were used to detect the expression and activity of MMP-2 and MMP-9. β-Actin was used as loading control.

To find out if DIM could inhibit FAK phosphorylation, we treated SMMC-7721 and MHCC-97H cells with DIM at the concentration of 10 μM, 15 μM and 20 μM in serum-free culture media for 48 h and analyzed the phosphorylation status of FAK. As shown in Fig 4A, we found that DIM decreased the expression of phosphorylated FAK, while the total FAK levels remain constant in both cell lines.

In order to determine if DIM could modulate MMP-2 and MMP-9 expression and activity, we treated SMMC-7721 and MHCC-97H cells with DIM at the concentration of 10 μM, 15 μM and 20 μM in serum-free medium. Western blotting and zymography were performed and the results showed that DIM decreased the expression and activity of MMP-2 and MMP-9 compared with control in both cell lines (Fig 4B).

### FAK inhibitor decreased the expression of MMP2/9 and suppressed the migration and invasion of HCC cells

**Figure 5:**
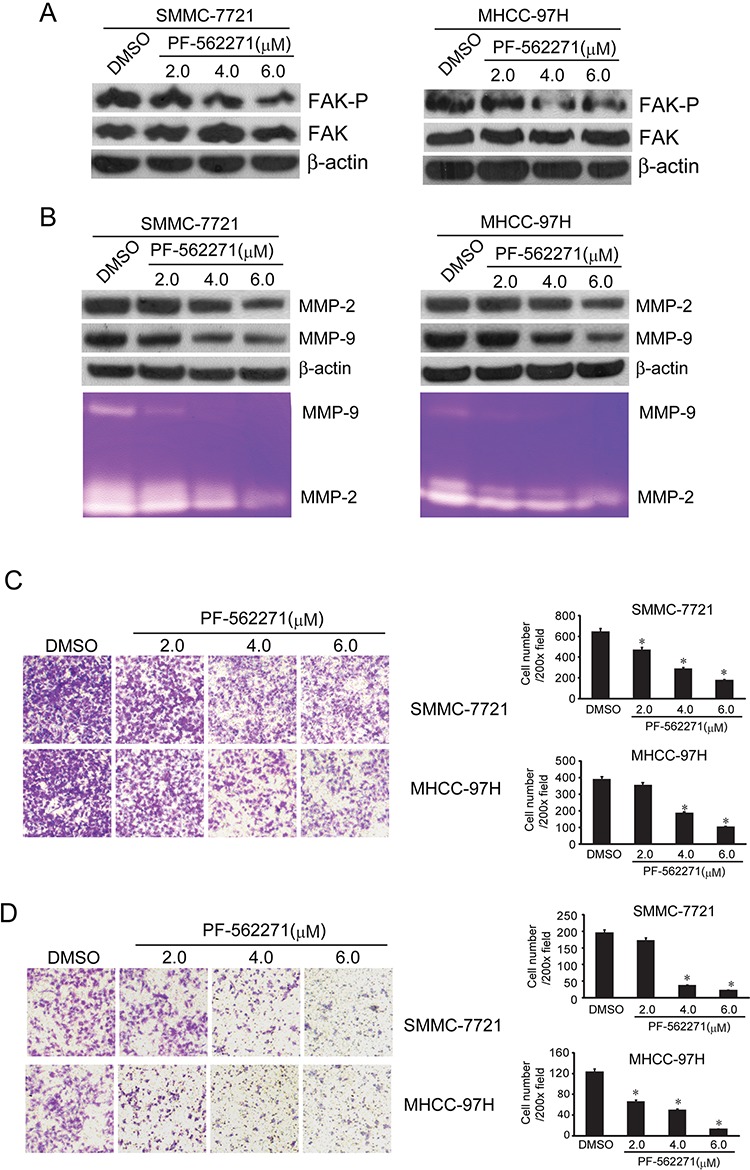
FAK inhibitor PF-562271 decreased the MMP2/9 levels and suppressed the invasion and metastasis of SMMC-7721 and MHCC-97H cells SMMC-7721 and MHCC-97H cells were treated with PF-562271 at 2, 4 and 6 μM in serum-free medium for 48 hours. **A.** FAK and the phosphorylated FAK (Y397) was analyzed by Western blotting. β-Actin was used as loading control. **B.** Western blotting and zymography were used to detect the expression and activity of MMP-2 and MMP-9. β-Actin was used as loading control. **C.** Migration tests were performed with transwell, SMMC-7721 and MHCC-97H (1 × 10^5^ cells per well) were seeded in insert in 200 μl no-serum medium containing different concentration of PF-562271 (2, 4 and 6 μM), 800 μl medium containing 5% FBS was added in bottom wells and cells were incubated for 16 hours. Cells were stained with Giemsa. **D.** Cells invasion was performed via transwell inserts with basement membrane extract. SMMC-7721 and MHCC-97H (1 × 10^5^ cells per well) were seeded in insert in 200 μl no-serum medium containing different concentration of PF-562271 (2, 4 and 6 μM), 800 μl medium containing 5% FBS was added in bottom wells and cells were incubated for 24 hours. Cells were stained with Giemsa. Values represent mean ± SD of three independent experiments. *P < 0.05, **P < 0.01, ***P < 0.001 compared with the untreated control (dose 0).

The above results indicated that DIM could inhibit the migration and invasion of HCC cells. At the same time it decreased the expression of phosphorylated FAK and MMP2/9. This implicated that DIM might inhibit cell migration and invasion through the regulation of FAK and MMP2/9. The FAK specific inhibitor PF-562271 inhibits the phosphorylation of FAK tyrosine 397 (Tyr397). We tested the inhibitory effect of PF-562271 and found that PF-562271, at the concentration of 2.0, 4.0 and 6.0 μM and treated for 48 hours, decreased the level of phosphorylated FAK without changing the total FAK in both cell lines. (Fig 5A),

In order to find out if PF-562271 could decrease MMP2/9 expression and inhibit cell migration and invasion, we treated the cells with PF-562271 at the concentration of 2.0, 4.0 and 6.0 μM for 48 hours, the expression and activity of MMP-2 and MMP-9 were analyzed by Western blotting and zymography. As showed in Fig 5B, PF-562271 decreased the expression and activity of MMP-2 and MMP-9 in a concentration-dependent manner in both cell lines. We also investigated the effect of PF-562271 on migration capability of HCC cells with transwell tests. As shown in Fig 5C, we found that PF-562271 significantly reduced the number of SMMC-7721 and MHCC-97H cells migrating to the lower chamber in a concentration-dependent manner.

The effect of PF-562271 on invasion, was shown in Fig 5D, PF-562271 significantly decreased the number of cells invading the lower surface of inserts compared with the control group in a concentration manner. The inhibition rate of invasion was 30%, 45%, 60% of control at 2.0, 4.0, 6.0 μM PF-562271 in SMMC-7721 and 45%, 55%, 85% of control in MHCC-97H (Fig 5D). These results illustrated that PF-562271, the specific inhibitor of FAK phosphorylation could decrease the invasive and migratory ability of tumor cells through inhibiting FAK pathway mediated MMP-2 and MMP-9 expression and activity.

### Silent FAK repressed the expression of MMP2/9 and suppressed the invasion of HCC cells by down-regulation of FAK

**Figure 6:**
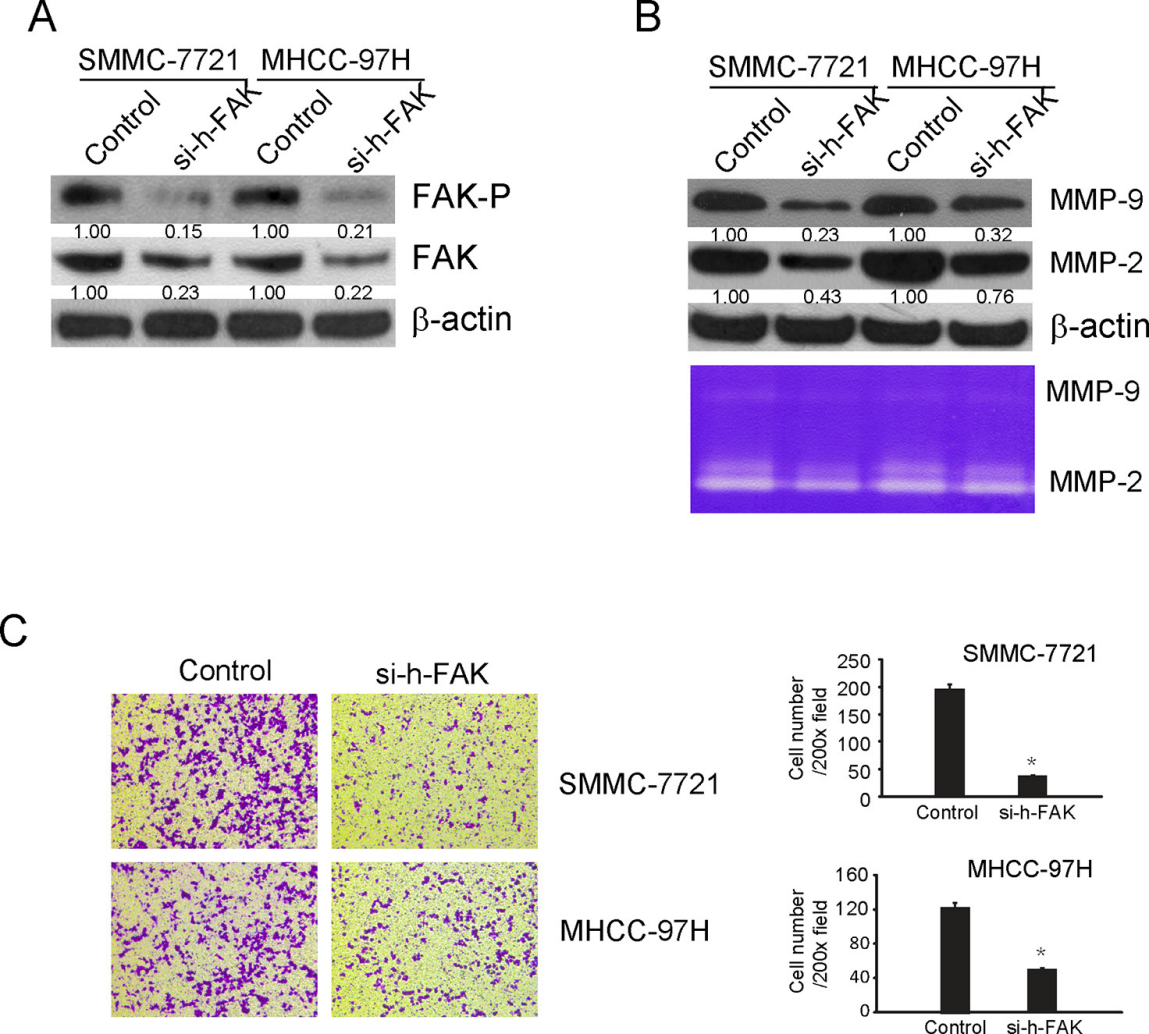
siRNA for FAK(si-h-FAK) down regulated the MMP2/9 levels and suppressed the invasion of SMMC-7721 and MHCC-97H cells by decreasing the levels of phosphorylated FAK and total FAK SMMC-7721 and MHCC-97H cells were plated in a 6-well plate (6 × 10^5^ cells/well) and transfected with FAK siRNA or control non-targeted siRNA. Forty-eight hours after transfection, cells were collected for western blotting to confirm the effects of siRNA and other tests. **A.** FAK and the phosphorylated FAK (Tyr397) were analyzed by Western blotting. β-Actin was used as loading control. **B.** Western blotting and zymography were used to detect the expression and activity of MMP-2 and MMP-9. β-Actin was used as loading control. **C.** Cells invasion was performed via transwell inserts with basement membrane extract. Cells were stained with Giemsa. Values represent mean ± SD of three independent experiments. *P < 0.05, **P < 0.01, ***P < 0.001 compared with the untreated control (dose 0).

In order to confirm the results concluded from above tests, siRNA for human FAK (si-h-FAK) were used to silent the expression of FAK. siRNA has the advantage of high specificity, high efficiency and easy operation. As shown in Fig 6A, 48 h after siRNA transfection, the level of phosphorylated FAK was decreased to 15% in SMMC-7721 and 21% in MHCC-97H compared with that in control. In addition, the expression of total FAK was also decreased. In order to find out if si-h-FAK could decrease MMP2/9 expression and inhibit cell invasion, the expression and activity of MMP-2 and MMP-9 were analyzed by Western blotting and zymography 48 h after transfection. As showed in Fig 6B, si-h-FAK decreased the expression and activity of MMP-2 and MMP-9 in both cell lines. The effect of si-h-FAK on cell invasion was shown in Fig 6C, si-h-FAK significantly decreased the number of cells invading the lower surface of inserts compared with the control group in a concentration dependent manner. The inhibition rate of invasion was 30% of control in SMMC-7721 and 45% of control in MHCC-97H (Fig 5C). These results illustrated that si-h-FAK, similar with PF-562271, could decrease the invasive ability of tumor cells through inhibiting FAK mediated MMP-2 and MMP-9 expression and activity.

### DIM inhibited cell proliferation, migration and invasion through repressing FAK pathway and associated elements induced by vitronectin

Vitronectin (VTN) is a multifunctional glycoprotein present in blood and in the extracellular matrix (ECM). It binds to the integrin receptor αvβ3, activates the integrins/FAK pathway and involves in the cell attachment, spreading and metastasis [24]. To further test whether DIM could inhibit metastasis and invasion through the inactivation of FAK pathway and downstream signal VTN, we used purified human VTN to activate FAK and induce cells invasion.

**Figure 7:**
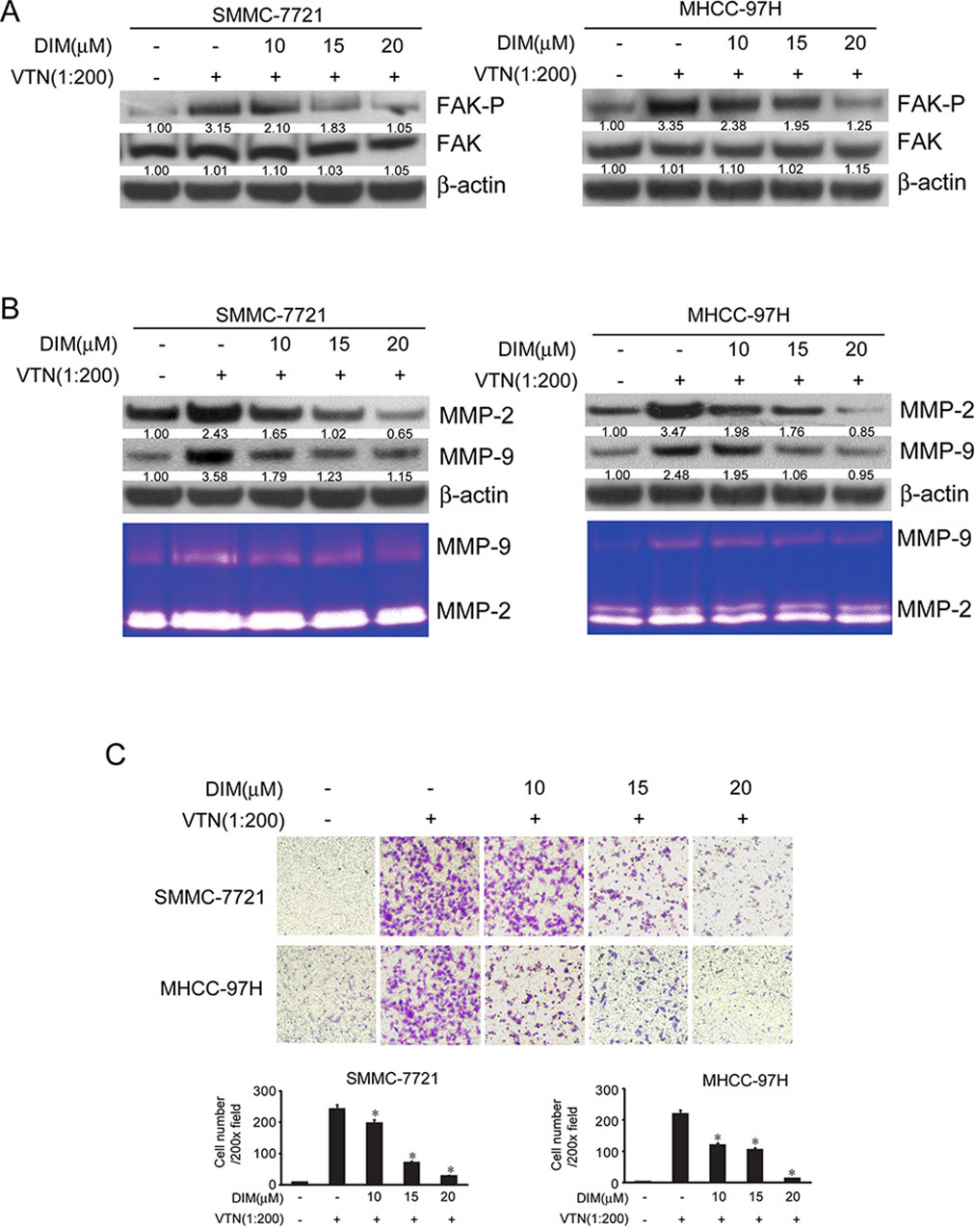
DIM inhibited cell migration and invasion through suppressing the activity of FAK pathway and MMP2/9 expression induced by vitronectin SMMC-7721 and MHCC-97H cells were treated with DIM (10, 15 and 20 μM) on VTN-coated or BSA-coated plate for 48 h. **A.** Western blotting analysis of cell lysates for FAK and FAP(Y397)-p, MMP-2 and MMP-9. **B.** Western blotting and zymography assay of MMP-2 and MMP-9. **C.** Cells invasion was performed via transwell inserts with basement membrane extract. SMMC-7721 and MHCC-97H (1 × 10^5^ cells per well) were seeded in insert in 200 μl no-serum medium containing different concentration of DIM (10, 15 and 20 μM), the bottom wells were loaded with 800 μl no-serum medium containing 5 ng/ml vitronectin or 5 ng/ml BSA and cells were incubated for 24 h. Cells were stained with Giemsa. Values represent mean ± SD of three independent experiments. *P < 0.05, **P < 0.01, ***P < 0.001 compared with the untreated control (dose 0).

In the present study, SMMC-7721 and MHCC-97H were treated with DIM (10, 15 and 20 μM) in VTN-coated or BSA-coated plate. After 48 h of treatment, cells were harvested and lysates were analyzed for FAK, phospho-FAK, MMP-2 and MMP-9. Cell conditioned medium were collected for the activity of MMP-2 and MMP-9 in zymography assay. As shown in Fig 7A, cells cultured in VTN coated plates had higher level of phosphorylated FAK compared with that of control, with no obvious change on total FAK. The level of phosphorylated FAK decreased in both cell lines with treatment of DIM. The expression of MMP-2 and MMP-9 and activation of MMP-2 and MMP-9 induced by VTN was also suppressed by the treatment of DIM (Fig 7B).

To examine whether DIM could suppress the invasion of cells induced by VTN, we performed invasion assays using transwell assay. VTN markedly increased invasion of SMMC-7721 (10 verse 230; *P* < 0.01) and MHCC-97H (15 verse 160; *P* < 0.01), however, DIM significantly decreased the numbers of cell invading through Matrigel-coated filters in a concentration-dependent manner (Fig 7C). These results suggest that DIM could significantly inhibit cell invasion induced by VTN through inactivation of integrin/FAK pathways.

### DIM inhibited the activity of FAK by increasing pTEN expression

**Figure 8:**
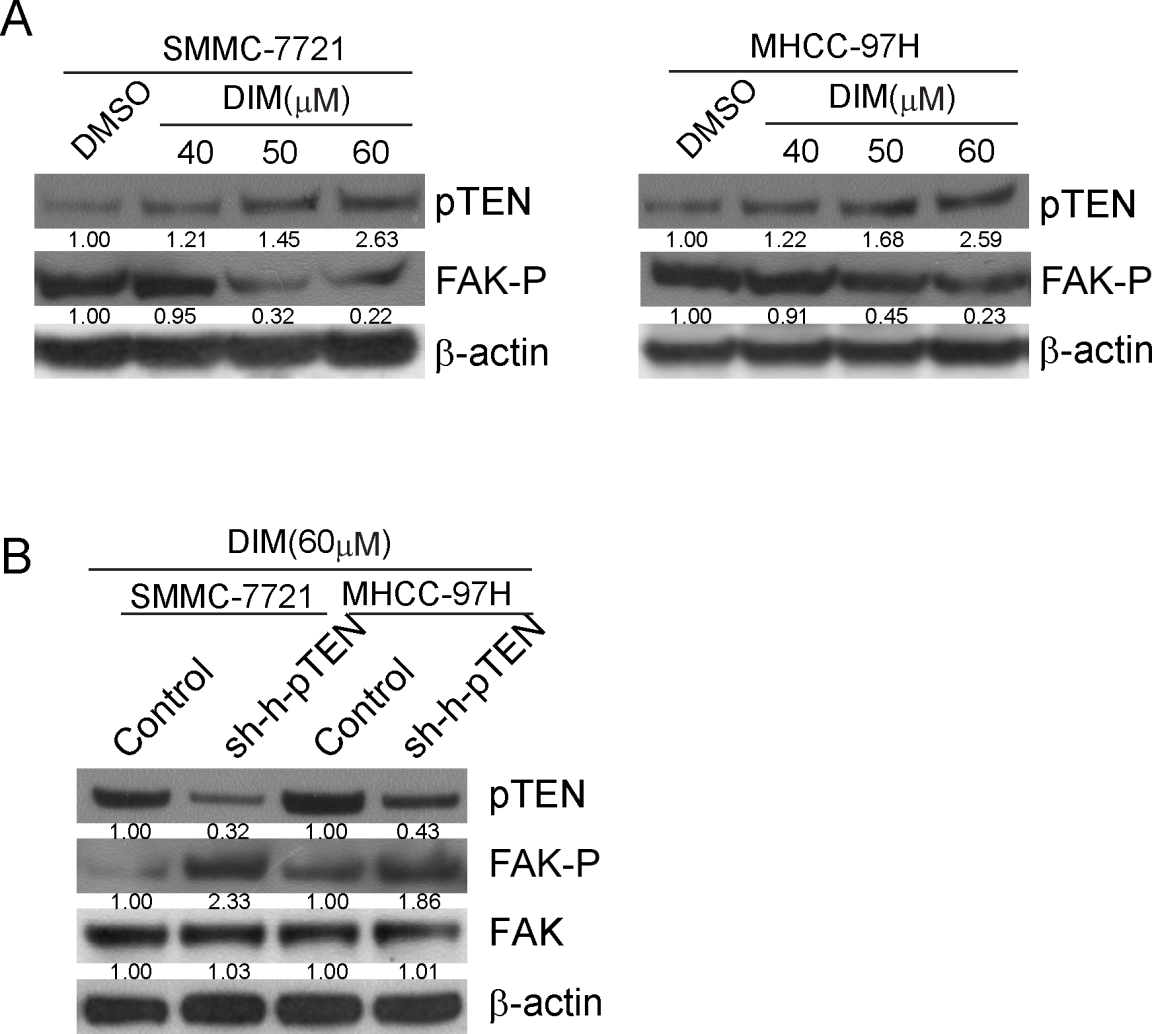
DIM inhibited the phosphorylation of FAK by up-regulating the expression of pTEN **A.** SMMC-7721 and MHCC-97H cells were treated with DIM at 40, 50 and 60 μM in full medium with 10% FBS and after 48 h of treatment, cells were harvested and protein extracts were analyzed for pTEN and FAK-p by Western blotting. **B.** SMMC-7721 and MHCC-97H cells were firstly transfected with pTEN siRNA or control and then treated with 60 μM DIM for 48 h. Cells were harvested and protein extracts were analyzed for pTEN and FAK-p by Western blotting.

PTEN, a lipid phosphatase, is known as an anti-oncogene which could directly inhibit FAK related pathways through directly dephosphorylation of FAK [25]. To find out whether DIM could change the expression of pTEN, we treated SMMC-7721 and MHCC-97H cells with DIM at 40, 50 and 60 μM in 10% FBS medium and after 48 h of total treatment, cells were harvested and protein extracts were analyzed for pTEN. The immunoblotting results illustrated that DIM obviously increased the expression of pTEN in a concentration-dependent manner compared with control in both cell lines (Fig 8A). To confirm that DIM could inhibit the phosphorylation of FAK through regulating the expression of pTEN, siRNA for human pTEN (si-h-pTEN) was used. As shown in Fig 8B, si-h-pTEN suppressed the over expression of pTEN induced by 60 μM DIM in SMMC-7721 and MHCC-97H cells. Additionally, the treatment of si-h-pTEN increased the level of phosphorylation of FAK inhibited by 60 μM DIM. These results revealed that pTEN might play a role in down-regulation of FAK by DIM.

### Oral administration of DIM inhibits lung metastasis of SMMC-7721 cells in BALB/c nude mice

**Figure 9:**
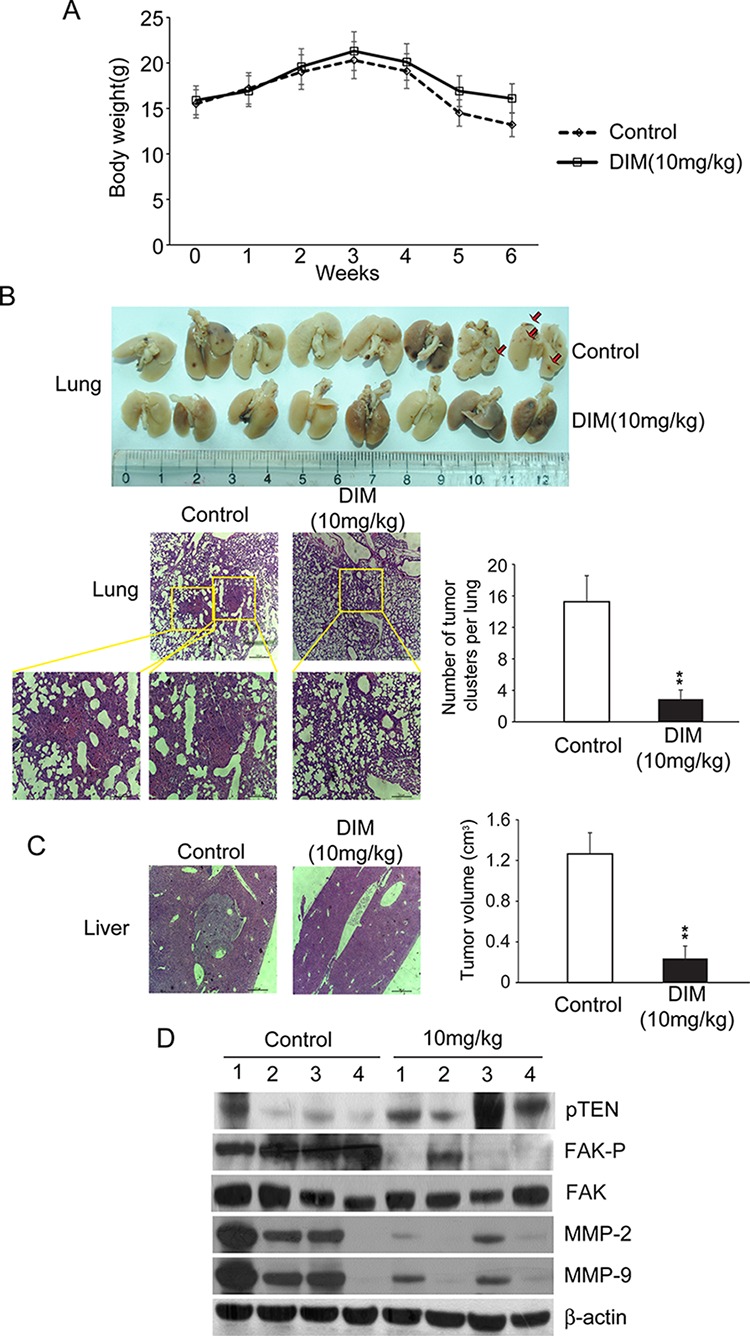
Oral administration of DIM inhibits lung metastasis of SMMC-7721 cells in BALB/c nude mice 5 × 10^5^ SMMC-7721 cells were injected into the liver of BALB/c nude mice to establish orthotopic liver cancer model. Nude mice were separated randomly into two groups, one group were treated with 10 mg/kg of DIM and the other were treated with control vehicle. After 6 weeks, nude mice were sacrificed. **A.** Line graph showing control and treated nude mice body weight (g) over a six weeks period. **B.** Lung metastasis was observed in the lungs of mice. Only a few metastases were found in mice treated with DIM. The number of lung metastatic nodules on the surface was counted; representative hematoxylin and eosin (HE) staining confirmed the development of tumor in lung tissue. High-power magnification images (400X) of the same field were showed in blow. **C.** The development of *in situ* tumors were observer in livers, the volume of tumors were calculated and compared with control. **D.** Protein samples from *in situ* tumors of livers were analyzed for FAK, FAP-p, pTEN, MMP-2 and MMP-9 by Western blotting. β-Actin was used as loading control.

To determine the effect of DIM on *in vivo* tumor growth and metastasis, SMMC-7721 cells were injected into the liver to establish an orthotopic liver cancer model. Nude mice were separated randomly into two groups, one group was treated with DIM and the other one was treated with control vehicle. After 6 weeks, nude mice were sacrificed and liver tumor volumes were measured. In the whole experiment, the mean body weights of the DIM treatment group were similar with control groups, except the last week. After 5weeks, the weights of nude mice from control group decreased more quickly than treatment group because of cancer cachexia(Fig 9A). As shown in Fig 9C, the average volume of tumor in nude mice treated by DIM were markedly smaller than the control group (*P* < 0.01). Histological analysis demonstrated that intrahepatic tumor nodules were found larger in control mice than that in mice treated by DIM. To further investigate the effect of DIM on SMMC-7721 metastasis *in vivo*, their lung were collected after sacrificed and the number of nodules on the surface was counted. The mean number of metastatic nodule on the surface of the lung was significantly decreased in mice treated by DIM, compared with control, (2.8 in DIM treated verse 15.2 in control, *P* < 0.01) (Fig 9B). Pathological analysis demonstrated larger and greater number of tumor nodules in control mice while DIM treated mice displayed metastatic tumors that are fewer and smaller in size (Fig 9B). The expression of FAK, p-FAK, pTEN, MMP-2 and MMP-9 were analyzed by Western blotting. Consistent with our *in vitro* results, the expression of phosphorylated FAK and MMP2/9 in DIM-treated mice were significantly decreased compared with that in control mice, and the pTEN expression was also increased by DIM (Fig 9D). Taken together, our results from both *in vitro* and *in vivo* assays illustrated that DIM can inhibit the formation and metastasis of hepatocellular carcinoma through FAK signaling pathway.

## DISCUSSION

HCC is a common malignancy in many countries [26]. The low survival rate of late stage HCC is largely due to its high rate of intrahepatic and extra-hepatic metastasis [27]. DIM, the predominant active product of phytochemical indole-3-carbinol (I3C) from vegetables of the family cruciferous [12], inhibits tumor formation in the early phase of tumor genesis, induces cancer cells apoptosis and suppresses cancer cells metastasis or invasion in multiple types of cancer [13–18]. So far, little is known about the tumor inhibitory effect of DIM on HCC. In the current research, we used two HCC cell lines SMMC-7721 and MHCC-97H that have high potential of malignant invasion to investigate the anti-tumor effect of DIM. We found that DIM inhibited the proliferation, migration and invasion of these two cell lines. We further demonstrated the role of DIM in suppressing tumor metastasis through a mouse liver cancer model.

FAK plays a central role in many types of cell events including proliferation, survival, apoptosis, migration and invasion [28, 29]. FAK has been shown to be very important in the development of liver tumors. Chen et al. found FAK and phospho-FAK Tyr397 were overexpressed in HCC samples and HCC cell lines. The elevated FAK and phospho-FAK Tyr397 were correlated with tumor stage and vascular invasion in hepatocellular carcinoma [2]. *In vitro* and *in vivo* tests show that up-regulating FAK promotes tumor metastasis [30–32]. In our study, we detected FAK and phospho-FAK Tyr397 in several HCC cell lines, and also found that FAK and phospho-FAK Tyr397 were overexpressed in SMMC-7721 and MHCC-97H cells that have higher invasive potential compared with other cell lines. Previously, some researchers found that inhibition of FAK phosphorylation by siRNA or FAK specific inhibitors could suppress HCC cell adhesion, migration and invasion [2, 33–35]. We investigated whether DIM inhibited HCC cell metastasis through down-regulation of FAK. When SMMC-7721 and MHCC-97H were treated with different concentration of DIM, we found a significantly decreased level of phosphorylated FAK Tyr397. In order to confirm the results of FAK inhibition, we used a FAK specific inhibitor PF-56227. PF-56227interacts with FAK in the adenosine triphosphate (ATP) binding pocket and blocks the catalytic activity of FAK [36]. When phospho-FAK Tyr397 were inhibited by PF-56227 in SMMC-7721 and MHCC-97H cells, the metastatic ability of these two cell lines decreased. Similar conclusions were obtained after suing si-h-FAK to confirm the results observed through using PF-56227. We suggested that DIM might inhibit the migration and invasion of hepatocellular carcinoma cells through FAK signaling pathway.

Activation and phosphorylation of FAK by ECM-integrins is an important phenomena for tumor cells to achieve migratory phenotype [29]. Vitronectin (VTN), a multifunctional glycoprotein present in blood and in the ECM, involves in the cell attachment, spreading and metastasis [24]. FAK is localized in focal contacts that becomes tyrosine phosphorylated and subsequently activated on integrin-mediated cell adhesion to several matrix proteins, including VTN [37]. The highly invasive human cancer cells expressing integrin receptor αvβ3 binds to VTN and generates a migratory phenotype through the activation of FAK signaling [38]. In our study, purified human VTN was used to investigate the relationship of VTN and the anti-metastasis effect of DIM. The results illustrated that VTN increased the expression of phospho-FAK Tyr397 in SMMC-7721 and MHCC-97H cells and promoted invasions of these two cell lines in transwell tests. When DIM was added, the high level of phospho-FAK Tyr397 induced by VTN decreased and invasive potential of two cell lines weaken as well. The results further confirmed that DIM inhibited the migration and invasion of these two cell lines through FAK pathway.

The invasive nature of malignant tumors has been associated with the ability of tumors to degrade extracellular matrix [39]. Matrix metalloproteinase (MMPs), a family of zinc-dependent endopeptidases, could degrade almost all ECM components and involved in the tumor metastatic cascade [20, 39]. MMP2/9 are the main components of MMPs and closely associated with migration and invasion in HCC [7, 40]. In our study, we found that MMP2/9 are overexpressed in SMMC-7721 and MHCC-97H cells compared to other cell lines. This might be related to the high potential of invasion [21]. When we treated SMMC-7721 and MHCC-97H cells with DIM, we found that DIM could decrease the expression level and activity of MMP2/9 in these two HCC cell lines. These results were consistent with other reports which confirmed that DIM could inhibit the migration of cancer cells through down-regulating MMPs, including those in thyroid cancer [41], breast cancer [42] and prostate cancer [17, 43]. Accumulated evidence suggested that FAK play an important role in the regulation of MMP2/9, over-activation of FAK could induce MMP2/9 expression and promote cells invasion [44, 45]. Down-regulation of FAK could inhibit HCC cell migration and invasion partly through down-regulating expressions and activations of both MMP-2 and MMP-9 [2]. In our research, PF-56227, the inhibitor of FAK, decreased the expression and activation of MMP2/9 through the inhibition of FAK phosphorylation in SMMC-7721 and MHCC-97H cells. In the test of cell invasion induced by VTN, the expression and activation of MMP2/9 increased and this effect might be induced by the activation of FAK. When DIM was added, the level of phosphorylated FAK Tyr397 and the expression and activation of MMP2/9 were suppressed. We suggested that DIM might suppress the expression and activation of MMP2/9 through inactivation of FAK and lead to the anti-metastasis. The results of our *in vivo* orthotopic liver cancer model demonstrated that DIM inhibited liver cancer growth and decreased its metastasis to lung. DIM also inhibited the level of phosphorylated FAK Tyr397 and decreased the expression of MMP2/9 in liver tumor tissues.

How did DIM regulate the phosphorylation of FAK? PTEN, known as an anti-oncogene, could directly inhibit FAK related pathways through directly de-phosphorylation of FAK [25]. Recently, Sarkar et al. found that DIM could up-regulate pTEN expression through down-regulating the expression of miR-221 and lead to the inhibition of cell proliferation and migration of pancreatic cancer cells [46]. In our study, we observed that DIM increased the expression of pTEN in both hepatocellular carcinoma cell lines, and si-h-pTEN could inhibit the effects of DIM on FAK phosphorylation. In *in vivo* tests, the expression of pTEN increased by addition of DIM. We speculated that pTEN might play a role in DIM regulation of FAK.

In summary, our data demonstrated that DIM could inhibit the proliferation, invasion and migration of hepatocellular carcinoma cells both *in vivo* and *in vitro*. These inhibitory effects are through increasing pTEN expression and inhibiting FAK phosphorylation leading to decreased MMP2/9 expression and activation. Since long-term exposure to DIM produced no observable toxicity and DIM is a less efficacious inducer of CYPs [47], DIM could be a promising anti-cancer drug in the future.

## MATERIALS AND METHODS

### Cell lines and cultures

HCC cell lines (HepG2, QGY-7701, Bel-7402, SMMC-7721 and MHCC-97H) were obtained from the Cell Bank of the Chinese Academy of Sciences (Shanghai, China) and cultured in Gibco^®^ RPMI Media 1640 with 10% fetal bovine serum(FBS), penicillin (100 U/ml) and streptomycin (100 mg/ml). All cells were grown as monolayer cultures and maintained in a humidified atmosphere of 5% CO_2_ in air at 37°C. Penicillin, streptomycin, RPMI media 1640 and FBS were purchased from Life Technologies, Inc. (Grand Island, NY).

### Reagents and antibodies

DIM was purchased from Sigma-Aldrich (St Louis, MO) and dissolved in DMSO to make a 100 mmol/L stock solution and stored at −20°C in multiple aliquots. PF-562271(N-methyl-N-{3-[({2-[(2-oxo-2, 3-dihydro-1H-indol-5-yl)amino]-5-(trifluoromethyl)pyrimidin-4-yl}amino) methyl]pyridin-2-yl}methane sulfonamide), was obtained from Selleckchem (Houston, TX) and dissolved in DMSO to make a 10 μmol/L stock solution and stored at −20°C. Vitronectin was obtained from Life Technologies, Inc. (Grand Island, NY) and dissolved in phosphate buffered saline with calcium and magnesium (0.90 mM CaCl_2_, 0.49 mM MgCl_2_, 2.67 mM KCl, 1.47 mM KH_2_PO_4_, 137.93 mM NaCl, 8.06 mM Na_2_HPO_4_) to make a 0.5 ug/ml stock solution. Antibodies against Phospo-FAK (Tyr397), FAK, MMP-2, MMP-9, pTEN, β-actin were purchased from Cell Signaling.

### Cell proliferation assay

Cell proliferation was assessed by WST-1 Cell Proliferation and Cytotoxicity Assay Kit (C0035, Beyotime Institute of Biotechnology, China). In brief, HepG2, Bel-7402, QGY-7701, SMMC-7721 and MHCC-97H were seeded in 96-well plates at 2.5 × 10^3^ cells/well and allowed to adhere for 24 h. Then cells were treated with increasing concentration of DIM (30 μM, 40 μM and 60 μM) in 1640 complete medium for 24 h, 48 h and 72 h. After treatment, WST-1 was added to each well and incubated for 2 h at 37°C. Absorbance was recorded at 450 nM. Inhibition of DIM on cell proliferation was calculated based on the absorbance ration between treatment and control.

### Cell adhesion assay

SMMC-7721 and MHCC-97H cells were treated with DIM at 30, 40 and 50 μM for 48 h. After that, Cells (5 × 10^5^ cells/well) treated by DIM were plated in 6-well culture dishes and allowed to adhere for 2.5 h. After that, medium with non-adhered cells was discarded and wells were gently washed twice with PBS to remove any loosely attached cells. Adhered cells were stained with 0.4% trypan blue solution (Sigma-Aldrich, St. Louis, MO) and counted. Adhered cells were counted and data expressed as percent decrease in adhered cell count for cells treated with DIM relative to control cells.

### Wound healing assay

Cell migration was analyzed by a wound healing assay [48]. In brief, SMMC-7721 and MHCC-97H cells were seeded into 6-well plates and allowed to confluent. A scratch wound in confluent monolayer was made using a pipette tip. After washing away all detached cells with PBS, the remaining cells were treated with DMSO or DIM in fresh complete medium. Photographs were taken at 48 h and 72 h after treatment.

### *In vitro* cell migration and invasion assay

In order to test the ability of cells migration, 6.5mm Transwell^®^ with 8.0 μm Pore Polyester Membrane Insert (Product #3464, Corning, Inc., Corning, NY) was used. In order to test the invasion ability of cells crossing through matrigel-coated filter, 6.5 mm Transwell^®^ with 8.0 μm Pore Polycarbonate Membrane Insert coated with Cultrex^®^ Basement Membrane Extract (BME) (Product#3432-001-01, Trevigen Inc.) were used. The procedure was performed as described previously [49]. Cells were starved for 18 h using no-serum medium and then 1 × 10^5^ cells per well were seeded in insert with 200 μl no-serum medium with or without DIM, and 800 μl of growth medium containing 5% FBS was added in bottom wells. Following a culture of 16 h, non-migrating cells were removed from the upper surface by wiping with a cotton swab. The membrane was fixed with 4% formaldehyde for 15 min at room temperature. Cells were stained with Giemsa for 25 min, and their numbers in 5 fields of each membrane were counted under an inverted microscope. The procedure of PF-562271 was the same as that of DIM, the no-serum medium containing different concentration of PF-562271 was loaded in inserts. For the co-treatment of vitronectin and DIM, the same procedures were used except that the bottom wells were loaded with no-serum medium containing 5 ng/ml vitronectin or 5 ng/ml BSA.

### Western blotting analysis

Whole cell lysate preparation and Western blotting analysis were performed as previously described [50]. Briefly, cells were pelleted by centrifugation at 320 ×g for 10 min and suspended in lysis buffer (20 mM Tris-HCl pH 7.4, 2 mM EDTA, 500 mM sodium orthovanadate, 1% Triton X-100, 0.1% SDS, 10 mM NaF, 10 mg/mL leupeptin, and 1 mM PMSF). Aliquots (20 mg) of the lysates were separated on a 4–12% SDS-polyacrylamide gel and transferred into a PVDF membrane (Millipore, USA). Blots were blocked for 2 h in blocking buffer (5% non-fat dry milk in PBST buffer (10 mM phosphate buffer, 2.7 mM KCl, 140 mM NaCl and 0.05% Tween 20, pH 7.4)) and incubated with primary antibodies (1:1000) overnight at 4°C. After washing with PBST, the appropriate HRP-conjugated secondary antibody (1:5000) was added to the preparation. The blot was incubated at 37°C for 1 h and developed using an enhanced chemiluminescence detection system (Beyotime Institute of Biotechnology, China).

### MMP detection

The activity of MMP-2 and MMP-9 was detected through gel zymography as described before [51]. Briefly, SMMC-7721 and MHCC-97H cells were seeded at a density of 5 × 10^4^ cells per well in 6-well culture dishes and allowed to adhere overnight. Then they were switched to serum free medium and incubated with different concentration of DIM, PF-562271 or left untreated for 48 h. Conditioned medium were harvested and centrifuged to remove any debris. According to the cell numbers in each well, the total protein concentration of the medium was adjusted to be equal. 10% SDS-PAGE containing 0.1% gelatin was used and the conditioned medium was resolved in non-reducing conditions. After electrophoresis, gels were incubated in re-naturation buffer (2.5% Triton X-100) for 1 h on an orbital shaker and switched to developing buffer (50 mMTris-HCl pH 8, 5 mM CaCl_2_, 0.02% NaN_3_) for 1 h followed by 24 h at 37°C with new developing buffer. Gels were then stained with Comassie blue (G-250) and destained with 30% methanol and 10% acetic acid.

### VTN coating and treatment

Because serum contains several kinds of ECM proteins, including VTN, therefore, experiments about VTN were performed under serum-free conditions. Serum-free medium was supplemented with ITS (insulin, transferrin, selenium; Life Technologies, Inc., Rockville, MD). For treatment with the combination of VTN and DIM, the 6-well cell culture plates were coated with 5 μg/ml VTN (PHE0011, Life Technologies, Inc. (Grand Island, NY))and control dishes were blocked with 0.1% BSA overnight at 4°C in PBS and then washed with PBS. SMMC-7721 and MHCC-97H cells at 2.5 × 10^3^ cells/well were allowed to adhere for 24 h. Then cells were treated with increasing concentration of DIM (30 μM, 40 μM and 50 μM) in 1640 serum-free medium for 48 h. After treatment, cells were collected for Western blot analysis, the conditioned medium were collected for gel zymography.

### Transfection of cells with siRNA for FAK or pTEN

SMMC-7721 and MHCC-97H cells were plated in a 6-well plate (6 × 10^5^ cells/well) and transfected with FAK or pTEN siRNA or control non-targeted siRNA (Guangzhou RiboBio Co., Ltd., China) using lipofectamine TM 2000 (11668-019, Life Technologies, Inc. Grand Island, NY)) according to the manufacturer's instructions. Forty-eight hours after transfection, cells were collected for Western blotting to confirm the effects of siRNA and other tests.

### *In vivo* lung metastasis experiments

HCC mouse model was created using SMMC-7721 cells. Briefly, SMMC-7721 cells at logarithmic growth phase were collected, washed three times with PBS and adjusted the cell number to 1 × 10^8^ cells/ml. BALB/c nude mice were anesthetized with isofluorane, put in the supine position, and limbs were fixed with rubber band in the thermostatic experimental board. After disinfection with 2% tincture of iodine and 75% alcohol, the skin and peritoneum were opened, left lobe of the liver was extruded out of abdominal cavity. Using a micro-syringe, 5 μl of cell suspension was injected into the left lobe of the liver. After injection, the pinhole was pressed immediately with sterile swab to stop bleeding. Then, the left lobe of liver was put back into the abdominal cavity and the abdominal wall was closed. A week later, nude mice with injection of HCC cells were separated randomly into two groups, one group received 1 mg/ml DIM (10 mg/kg.d) which was suspended in drinking water with an emulsifier, 0.5% of ethoxylated castor oil (Sigma, St. Louis, MO). The other group received water with 0.5% of ethoxylated castor oil as a vehicle control. Mice received a freshly made solution of DIM in their drinking water every 2 days. After 6 weeks, mice were sacrificed, tumors were removed and weighted. One part of tumors were frozen in liquid nitrogen for future analysis, the left, livers and lungs were collected and fixed in formalin, embedded into paraffin. Consecutive sections were made for every block of lung and liver tissue and stained with hematoxylin and eosin. The number of lung metastases was counted and evaluated independently by two pathologists.

### Statistical analysis

The data were presented as the means plus/minors standard deviation. SPSS 11.0 software was used for statistical analysis. The data for time and dosage effects were analyzed using two-way ANOVA. When appropriate, data were analyzed using one-way analysis of variance (ANOVA). The priori significance level was set at *P* < 0.05.
